# Supplementary material for: Production of Primary Metabolites by Rhizopus stolonifer, Causal Agent of Almond Hull Rot Disease
Source: Molecules. 2022 Oct 24;27(21):7199. doi: 10.3390/molecules27217199 (PMC9657676; doi:10.3390/molecules27217199)
Supplement: Supplementary file 1 [file molecules-27-07199-s001.zip › molecules-1936507-supp-done.pdf]

Supplementary Data

Production of primary metabolites by *Rhizopus stolonifer*, causal agent of almond hull rot disease

Anjali Zaveri [1*], Jacqueline Edwards [1,2] and Simone Rochfort [1,2]

[1] School of Applied Systems Biology, La Trobe University, Bundoora, VIC 3083, Australia
[2] Agriculture Research Victoria, 5 Ring Road, Bundoora, VIC 3083, Australia

Table S2. Results of ANOVA-simultaneous component analysis (ASCA) of ^{1}H NMR spectra of fumaric acid, lactic acid and ethanol production and sugar & nitrogen metabolization. Effect describes the relative influence of each variable (Isolates, Nitrogen and Days) on each spectrum. P-value is derived from permutation testing (100 permutation).

**Fumaric acid**				
**Variable**	**PCs**	**Cum Eigen Val**	**Effect**	**P-value**
Isolates	1	0.02	2.43	0.01
Sugar	1	0.20	18.65	0.01
Nitrogen	1	0.00010	0.01	0.24
Days	1	0.02	2.55	0.01
(Isolates) x (Sugar)	1	0.08	7.82	1
(Isolates) x (Nitrogen)	1	0.004	0.40	1
(Isolates) x (Days)	1	0.02	2.46	1
(Sugar) x (Nitrogen)	1	0.03	3.50	1
(Sugar) x (Days)	1	0.05	4.67	1
(Nitrogen) x (Days)	1	0.005	0.49	1
(Isolates) x (Sugar) x (Nitrogen)	1	0.071	7.14	0.01
(Isolates) x (Sugar) x (Days)	1	0.056	5.55	0.01
(Isolates) x (Nitrogen) x (Days)	1	0.023	2.29	0.2
(Sugar) x (Nitrogen) x (Days)	1	0.015	1.53	0.52
Mean			0	
Residuals			40.48	

**Lactic acid**				
**Variable**	**PCs**	**Cum Eigen Val**	**Effect**	**P-value**
Isolates	1	0.27	26.54	0.01
Sugar	1	0.01	0.46	0.01
Nitrogen	1	0	0.02	0.61
Days	1	0.02	2.23	0.01
(Isolates) x (Sugar)	1	0.01	0.77	1
(Isolates) x (Nitrogen)	1	0	0.04	1
(Isolates) x (Days)	1	0.06	5.61	1
(Sugar) x (Nitrogen)	1	0	0.31	1
(Sugar) x (Days)	1	0.01	0.55	1
(Nitrogen) x (Days)	1	0	0.09	1
(Isolates) x (Sugar) x (Nitrogen)	1	0	0.42	0.01
(Isolates) x (Sugar) x (Days)	1	0.02	2.23	0.01
(Isolates) x (Nitrogen) x (Days)	1	0	0.18	1
(Sugar) x (Nitrogen) x (Days)	1	0	0.45	0.06
Mean	-	-	0	-
Residuals	-	-	60.1	-

**Ethanol**				

Variable	PCs	Cum Eigen Val	Effect	P-value
Isolates	1	0.02	2.14	0.01
Sugar	1	0.27	25.21	0.01
Nitrogen	1	0	0.02	0.71
Days	1	0.23	22.07	0.01
(Isolates) x (Sugar)	1	0.04	4.67	1
(Isolates) x (Nitrogen)	1	0.01	0.98	1
(Isolates) x (Days)	1	0.02	1.92	1
(Sugar) x (Nitrogen)	1	0.03	3.06	1
(Sugar) x (Days)	1	0.07	6.47	1
(Nitrogen) x (Days)	1	0	0.27	1
(Isolates) x (Sugar) x (Nitrogen)	1	0.04	3.63	0.01
(Isolates) x (Sugar) x (Days)	1	0.04	3.78	0.01
(Isolates) x (Nitrogen) x (Days)	1	0.01	1.14	0.01
(Sugar) x (Nitrogen) x (Days)	1	0.01	1.34	0.01
Mean	-	-	0	-
Residuals	-	-	23.3	-

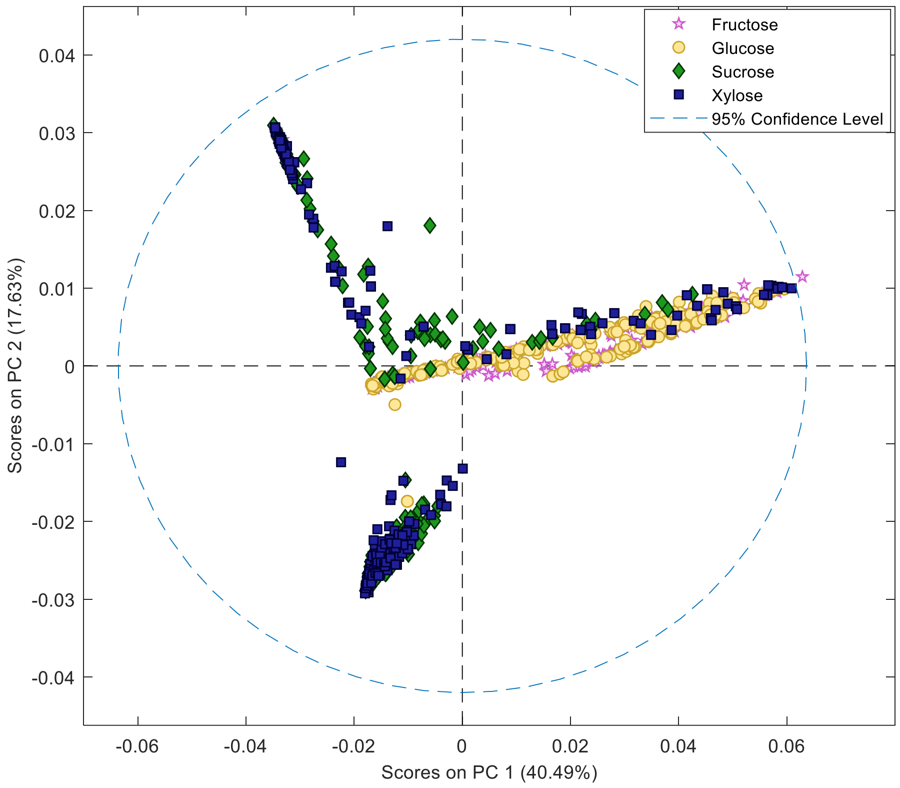

Figure S1. PCA score of ^{1}H NMR data of *in-vitro Rhizopus stolonifer* isolates.

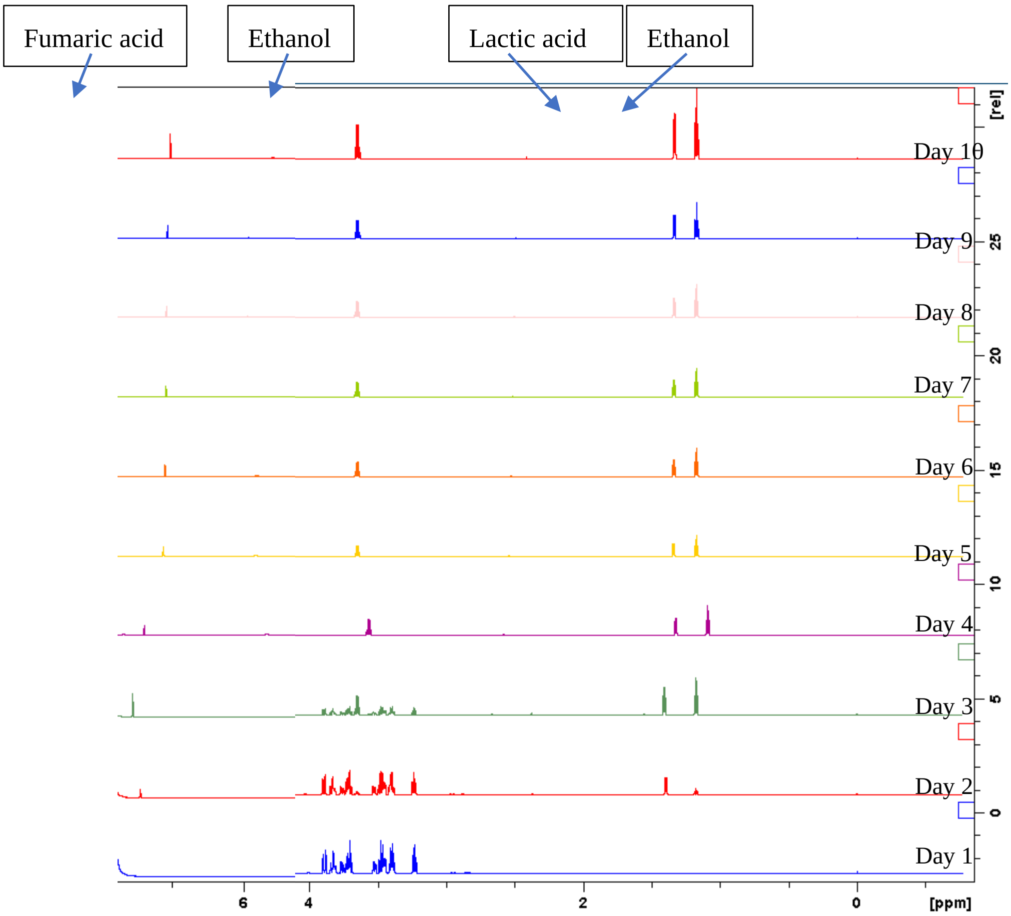

Figure S2. ^{1}H NMR spectra of in-vitro 19A-0030 glucose + asparagine samples showing resonances associated with major metabolites fumaric acid, lactic acid and ethanol over period of 10-day.

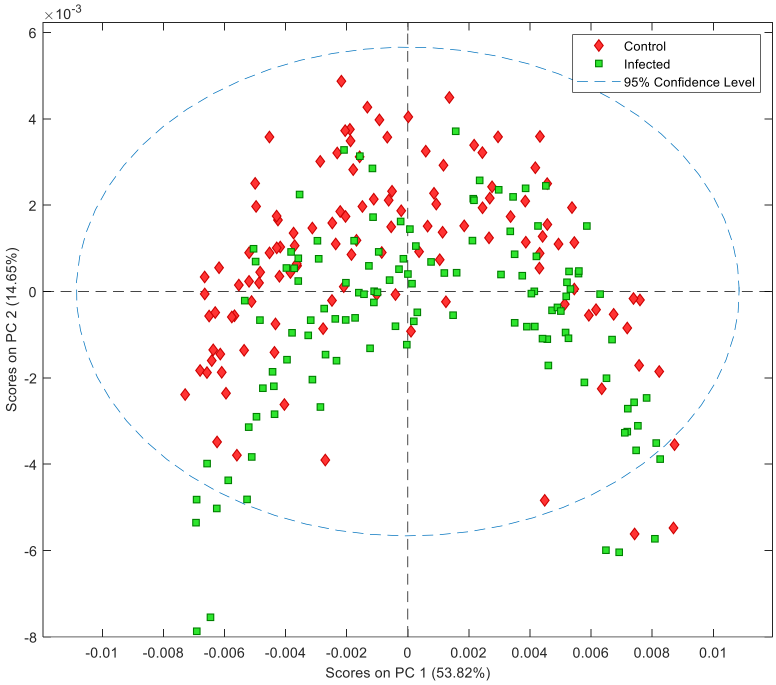

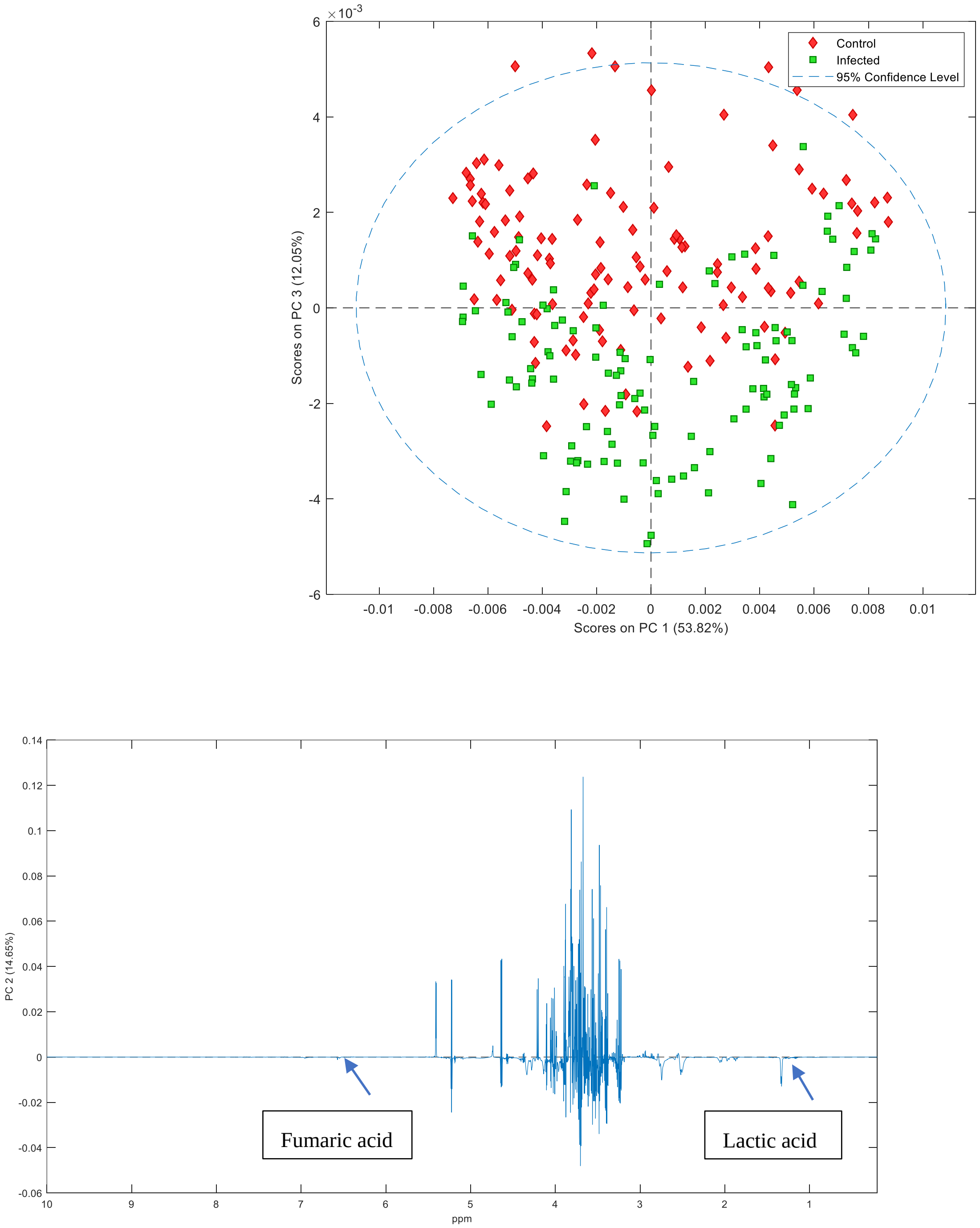

Figure S3. PCA score of ^{1}H NMR data of infected hull samples. (a) PCA scores plot showing separation of control and infected hull samples (b) loadings plot of P2 variable indicates that the lactic acid and fumaric acid are elevated under infected hull samples.

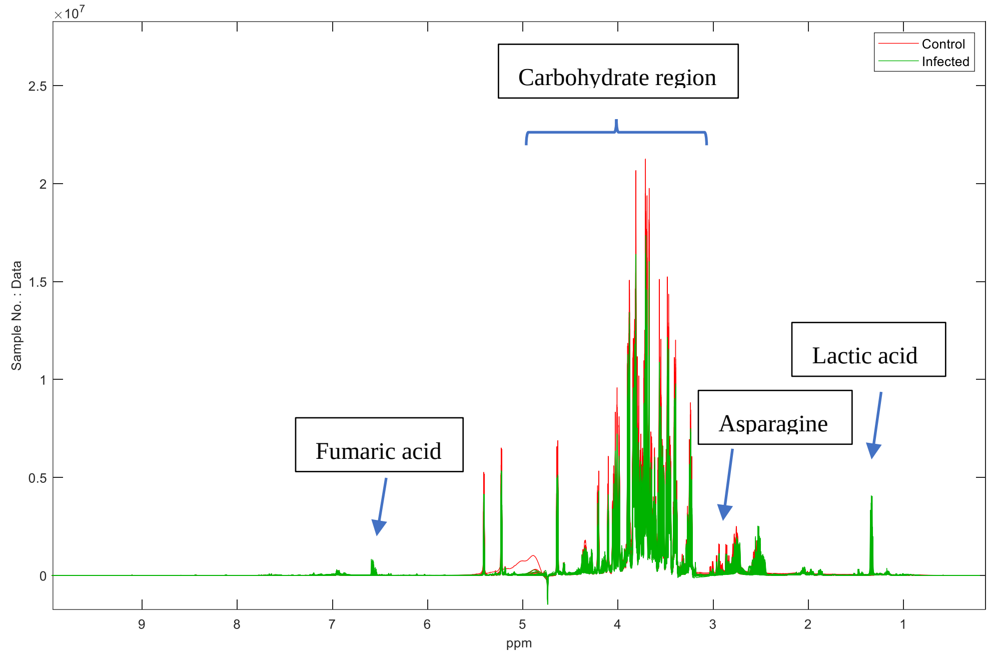

Figure S4. Comparison of control and infected Nonpareil hull components based on loadings plot: components of interest have been identified in infected almond hull composition such as glucose, sucrose, fructose, xylose, asparagine, fumaric acid and lactic acid. overlap of control and infected spectra showing elevated level of asparagine, glucose (δ 4.51ppm, δ 5.2ppm), fructose (δ 4.1 ppm), sucrose (δ 5.4 ppm) and xylose (δ 5.36 ppm). Elevated level of fumaric acid (δ 6.51 ppm) and lactic acid (δ 1.30 ppm) in infected samples.

R1

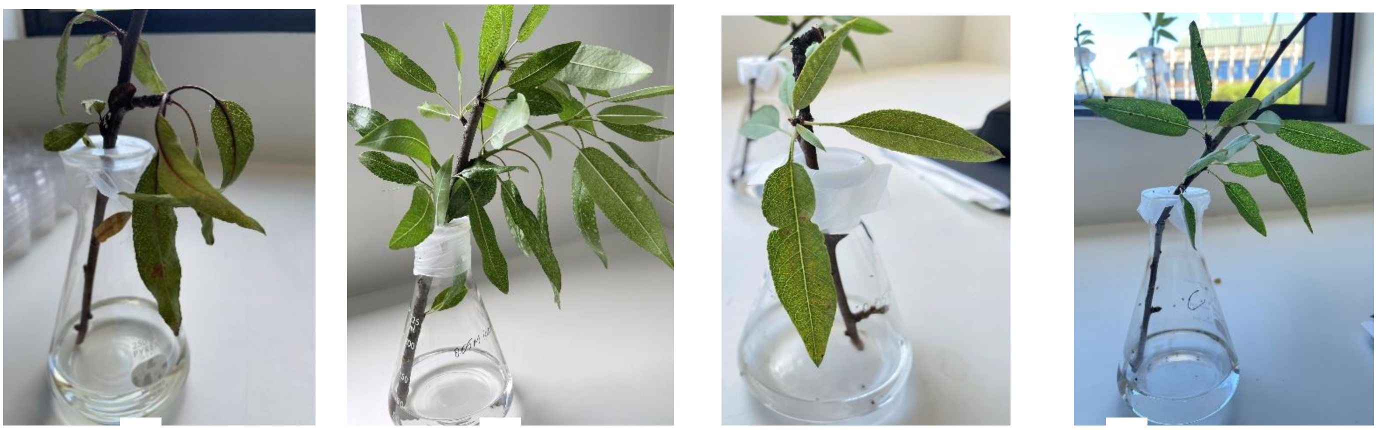

a **b** **c** **d**

R2

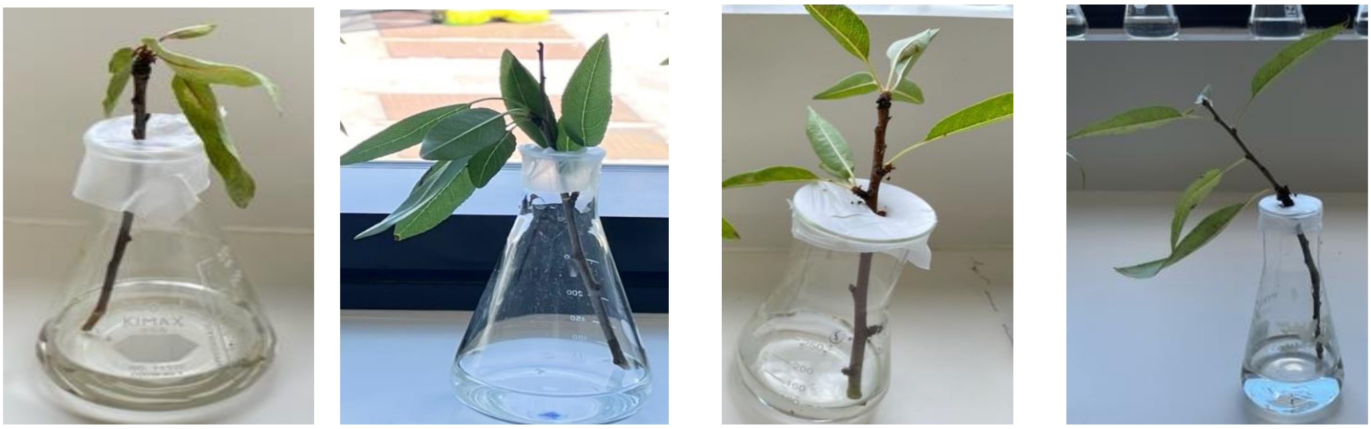

a **b** **c** **d**

R3

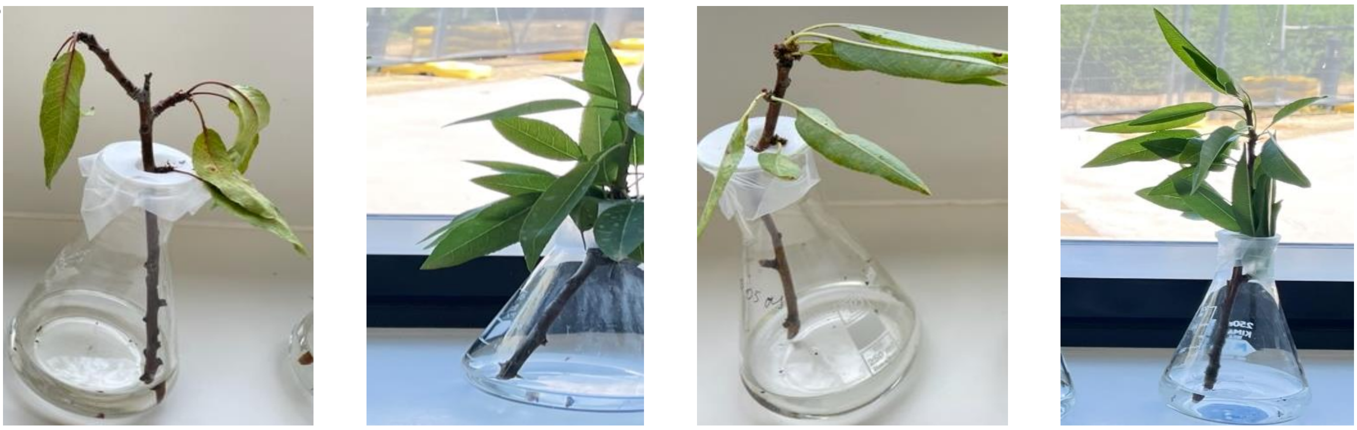

a **b** **c** **d**

(Symptoms after 24 hours)

R1

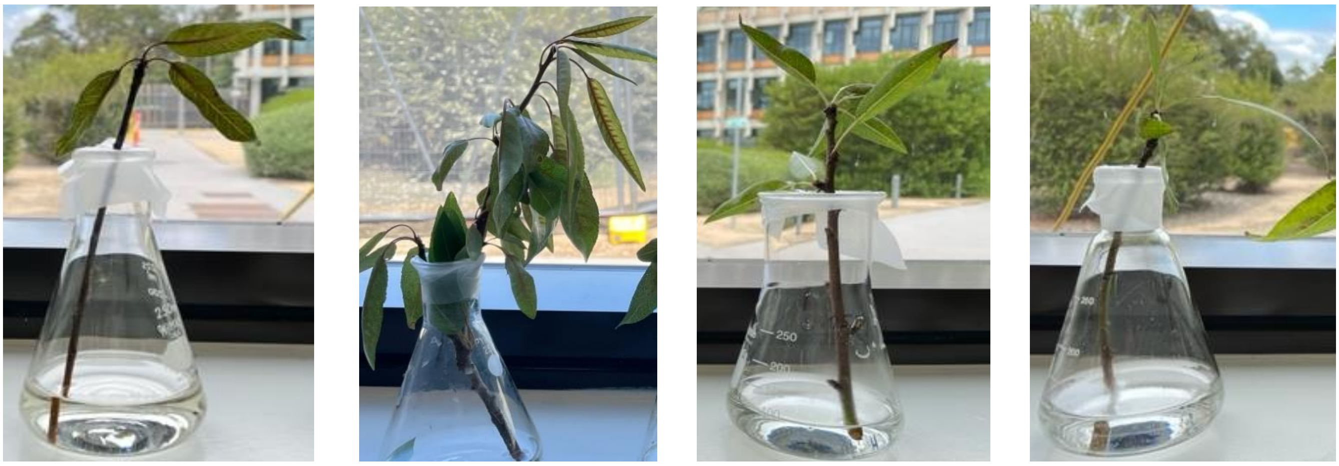

a **b** **c** **d**

R2

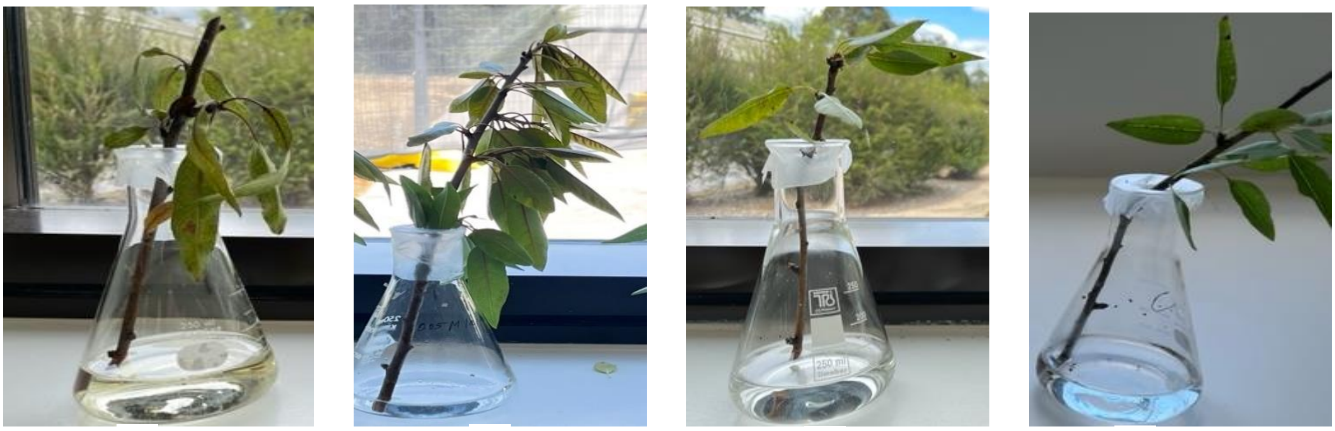

a **b** **c** **d**

(Symptoms after 48 hours)

R1

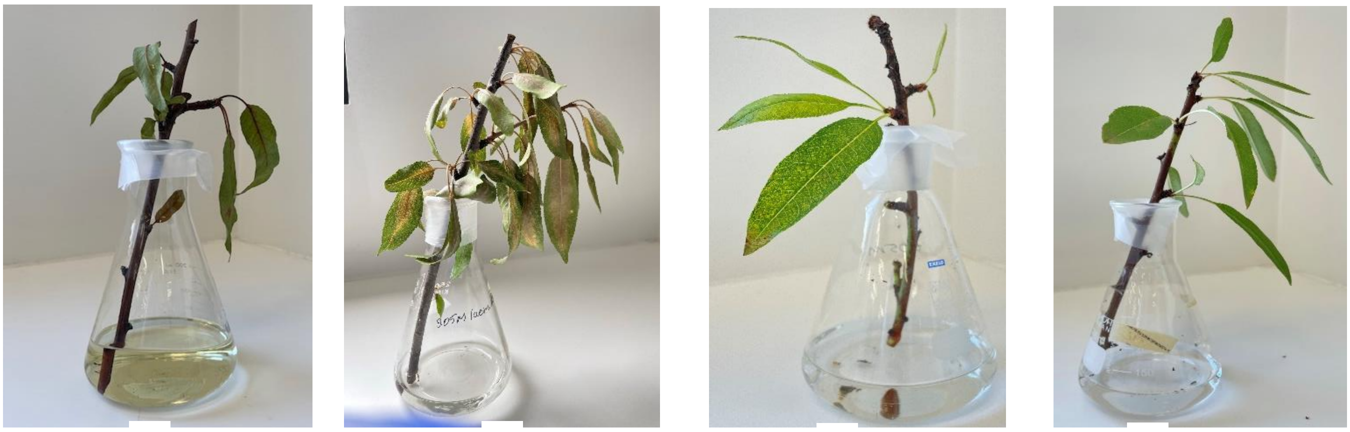

a **b** **c** **d**

R2

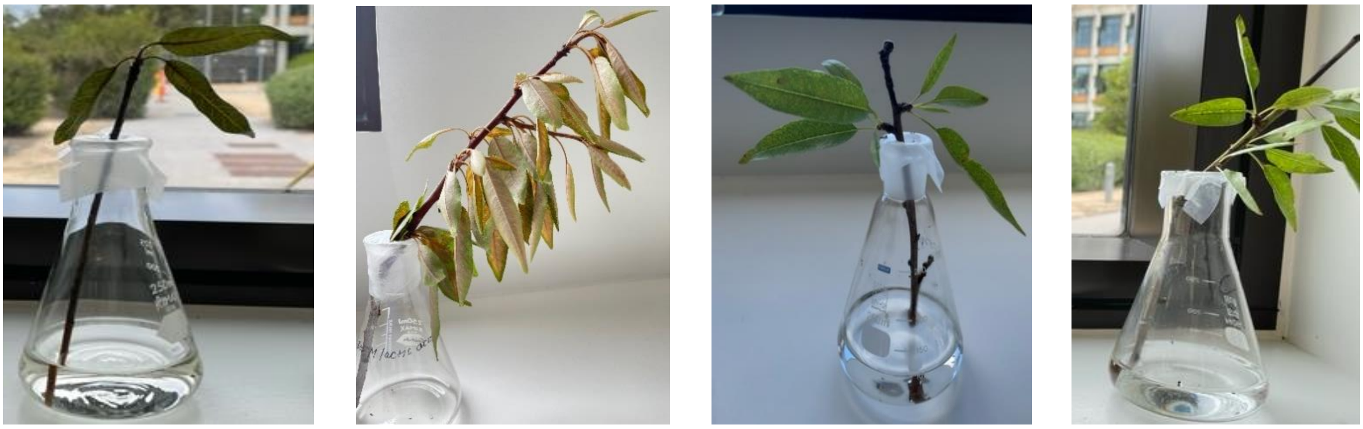

a **b** **c** **d**

R3

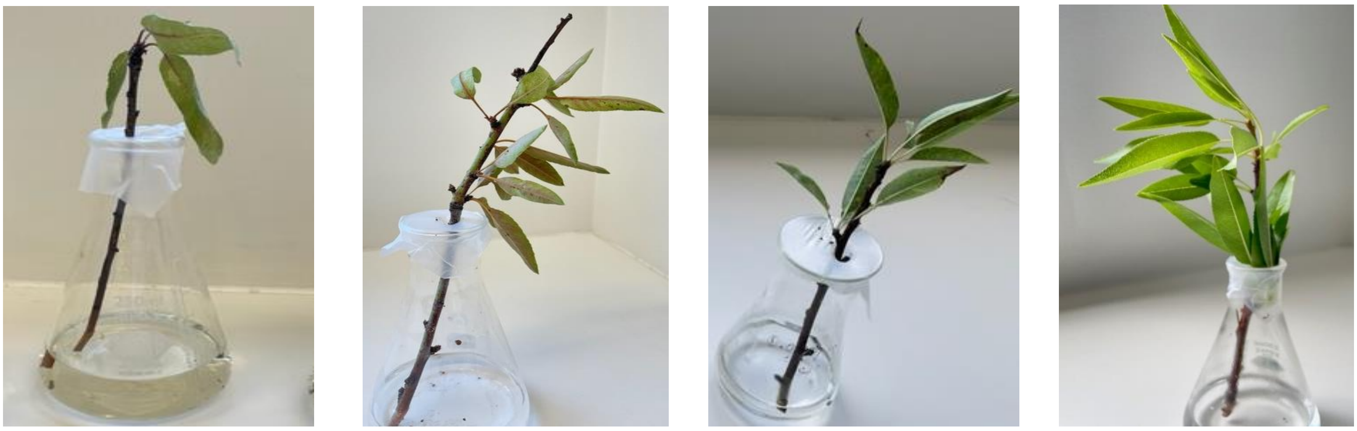

a b c d

(Symptoms after 72 hours)

Figure S5. Metabolite symptoms on almond shoots after 24 and 72-hour (a) Fumaric acid (b) Lactic acid (c) Ethanol (d) Control (water). *R1=replicate 1, R2= replicate 2, R3= replicate 3.

Table S3. Results of ANOVA-simultaneous component analysis (ASCA) of ^{1}H NMR spectra of infected hull samples. Effect describes the relative influence of each variable (Replicates and Treatment) on each spectrum. P-value is derived from permutation testing (100 permutation). NB.

All variable	PCs	Cum Eigen Val	Effect	P-value
Infection status	1	0.99	14.16	0.01
Treatment	3	0.44	6.34	0.01
(Uninfected /infected) x (Treatment)	3	0.25	3.58	0.01
Mean			0	
Residuals			75.92	
**All variable**	**PCs**	**Cum Eigen Val**	**Effect**	**P-value**

**Glucose**				
**Variable**	**PCs**	**Cum Eigen Val**	**Effect**	**P-value**
Infection status	1	0.02	2.39	0.01
Treatment	1	0.12	12.01	0.01
(Uninfected /infected) x (Treatment)	1	0.05	5.42	0.01
Mean			0	
Residuals			80.18	

**Fructose**				
**Variable**	**PCs**	**Cum Eigen Val**	**Effect**	**P-value**
Infection status	1	0.16	16.06	0.01
Treatment	1	0.15	14.96	0.01
(Uninfected /infected) x (Treatment)	1	0.07	7.08	0.01
Mean			0	
Residuals			61.90	

**Sucrose**				
**Variable**	**PCs**	**Cum Eigen Val**	**Effect**	**P-value**
Infection status	1	0.42	41.86	0.01
Treatment	1	0.04	3.64	0.01

(Uninfected /infected) x (Treatment)	1	0.02	2.13	0.01
Mean			0	
Residuals			52.37	

**Xylose**				
**Variable**	**PCs**	**Cum Eigen Val**	**Effect**	**P-value**
Infection status	1	0.03	4.96	0.01
Treatment	1	0.06	1.53	0.29
(Uninfected /infected) x (Treatment)	1	0.03	2.96	0.04
Mean			0	
Residuals			90.56	

**Asparagine**				
**Variable**	**PCs**	**Cum Eigen Val**	**Effect**	**P-value**
Infection status	1	0.03	3.19	0.01
Treatment	1	0.06	6.42	0.01
(Uninfected /infected) x (Treatment)	1	0.03	2.97	0.06
Mean			0	
Residuals			87.41	

**Fumaric acid**				
**Variable**	**PCs**	**Cum Eigen Val**	**Effect**	**P-value**
Infection status	1	0.14	14.46	0.01
Treatment	1	0.04	4.67	0.01
(Uninfected /infected) x (Treatment)	1	0.02	2.82	0.03
Mean			0	
Residuals			78.047	

**Lactic acid**				
**Variable**	**PCs**	**Cum Eigen Val**	**Effect**	**P-value**
Infection status	1	0.16	16.19	0.01
Treatment	1	0.01	1.15	0.27
(Uninfected /infected) x (Treatment)	1	0.02	1.67	0.15
Mean			0	
Residuals			80.99	
